# A Specific Blood Signature Reveals Higher Levels of S100A12: A Potential Bladder Cancer Diagnostic Biomarker Along With Urinary Engrailed-2 Protein Detection

**DOI:** 10.3389/fonc.2019.01484

**Published:** 2020-01-09

**Authors:** Ayssar A. Elamin, Saskia Klunkelfuß, Susanne Kämpfer, Wulf Oehlmann, Matthias Stehr, Christopher Smith, Guy R. Simpson, Richard Morgan, Hardev Pandha, Mahavir Singh

**Affiliations:** ^1^LIONEX Diagnostics and Therapeutics GmbH, Brunswick, Germany; ^2^Department of Oncology, Faculty of Health and Medical Sciences, University of Surrey, Guildford, United Kingdom; ^3^Institute of Cancer Therapeutics, Faculty of Life Sciences, University of Bradford, Bradford, United Kingdom

**Keywords:** bladder, cancer, microarray, S100A12, plasma, EN2, lateral flow, urine

## Abstract

Urothelial carcinoma of the urinary bladder (UCB) or bladder cancer remains a major health problem with high morbidity and mortality rates, especially in the western world. UCB is also associated with the highest cost per patient. In recent years numerous markers have been evaluated for suitability in UCB detection and surveillance. However, to date none of these markers can replace or even reduce the use of routine tools (cytology and cystoscopy). Our current study described UCB's extensive expression profile and highlighted the variations with normal bladder tissue. Our data revealed that *JUP, PTGDR, KLRF1, MT-TC*, and *RNU6-135P* are associated with prognosis in patients with UCB. The microarray expression data identified also *S100A12, S100A8*, and *NAMPT* as potential UCB biomarkers. Pathway analysis revealed that natural killer cell mediated cytotoxicity is the most involved pathway. Our analysis showed that S100A12 protein may be useful as a biomarker for early UCB detection. Plasma S100A12 has been observed in patients with UCB with an overall sensitivity of 90.5% and a specificity of 75%. S100A12 is highly expressed preferably in high-grade and high-stage UCB. Furthermore, using a panel of more than hundred urine samples, a prototype lateral flow test for the transcription factor Engrailed-2 (EN2) also showed reasonable sensitivity (85%) and specificity (71%). Such findings provide confidence to further improve and refine the EN2 rapid test for use in clinical practice. In conclusion, S100A12 and EN2 have shown potential value as biomarker candidates for UCB patients. These results can speed up the discovery of biomarkers, improving diagnostic accuracy and may help the management of UCB.

## Introduction

Bladder cancer (- urothelial carcinoma of the urinary bladder, UCB) is the fourth and ninth most common cancer in men and women, respectively (1, 2). The global prevalence of UCB in Europe and North America has been estimated at 2.7 million (1, 2). UCB leads to significant mortality, with a survival rate of just 47–57% when linked to muscle-invasive disease (3). In addition to its effect on UCB patients, the disease poses a significant economic burden on healthcare systems with a mean treatment and monitoring cost of ~200,000 USD per patient, rendering it the most expensive of all tumors to treat (4).

Generally, after haematuria, most patients experience a similar clinical process (5, 6). There were also several biomarkers used for diagnostic and monitoring purposes, but no marker has yet been shown to reduce the need for cystoscopy (6–11). This is especially problematic given the high recurrence rate, because of which lifelong surveillance is needed to detect any recurrence as early as possible (10). The development of reliable, non-invasive tests could therefore improve not only the UCB diagnosis itself but also the quality of life for patients with a disease history, and in this regard the detection of biomarkers in bodily fluids has shown a high potential (4–8, 10). The US Food and Drug Administration (FDA) has approved only a few urine-based tests, and there are currently no blood based tests (6, 10). Therefore, more novel biomarkers are urgently needed to detect UCB in general and especially in high-risk populations where the disease prevalence appears to be high (12, 13). The transcription factor Engrailed-2 (EN2) was previously shown to be a specific and potentially sensitive marker for bladder and prostate cancer (14–20). Using a standard ELISA method, we have previously shown that urinary EN2 could be detected and used as a UCB diagnosis biomarker, even in the early and non-invasive stages of the disease (15).

In addition to EN2, we have also investigated the diagnostic potential of S100 protein family members. The S100 family consists of 25 members, the expression of which have only been described in vertebrates (21). These proteins are characterized by a low molecular weight (9–13 kDa) and two Ca^2+^ binding sites in the form of EF-hands, one of which is unconventional (N-terminal) and has 100 times higher Ca^2+^ affinity than the canonical binding site in C-terminal. The S100 family members differ in length and sequence of the hinge region between the binding sites as well as the extension at C-terminal following the C-terminal EF-hand (22). The functions of S100 Proteins range from controlling protein phosphorylation, enzyme activity and transcription factors over the dynamics of cytoskeleton constituents, Ca^2+^ homeostasis and cell growth, and differentiation to an involvement in the inflammatory response (22, 23). They also mediate proinflammatory activity through binding the receptor for advanced glycation end-products (RAGE) on endothelial cells as well as recruitment of monocytes (23–27). Among the S100 group, S100A12, and S100P are unusual in that the coding gene can be found in the human genome but not in the mouse genome (28–30). S100A12 was recently shown to bind to CD36, a class B scavenger receptor, and this binding mediated translocation of CD36 to the membrane and where it can regulate lipid transport by direct interaction (31). Altered S100 protein levels have been linked to a variety of diseases, including cancer, neurodegenerative disorders, immune disorders, and inflammatory conditions (29). S100 genes also were shown to have roles in UCB progression and tumorigenesis (32). S100A8 and S100A9 were found to be increased in UCB patients and their expression related to stage and grade of the tumor (33). S100A12 RNA expression was shown to be increased in the tumors of transitional cell carcinoma (TCC) patients (34), and another study found that the urinary canine S100A8/A9 concentration relative ratio to S100A12 concentration maybe useful as a marker for canine TCC (35).

The main aim of this study was to assess the changes in blood gene expression in UCB patients and to identify genes serving as biomarkers for UCB diagnosis and progression. We identified elevated expression of the human S100A12 as a bladder cancer-enriched gene that is potentially a sensitive and specific diagnostic biomarker for UCB. Motivated by the fact that there is rapid growth in the demand for point-of-care tests based on lateral flow assays with high sensitivity, specificity and low cost, we also developed a lateral flow rapid test for detection of EN2 in urine samples. More than one hundred clinical samples were used to validate the rapid test which exhibited high sensitivity and specificity.

## Materials and Methods

### Ethics Statement

All patients and healthy volunteers participating in the study gave written informed consent for sample donation and the collection. The protocol was approved by the local ethical committee of Faculty of Health and Medical Sciences, University of Surrey (Ref. 3/LO/0739).

### Specimen, Data Collection, and Study Design

**Figure 1:**
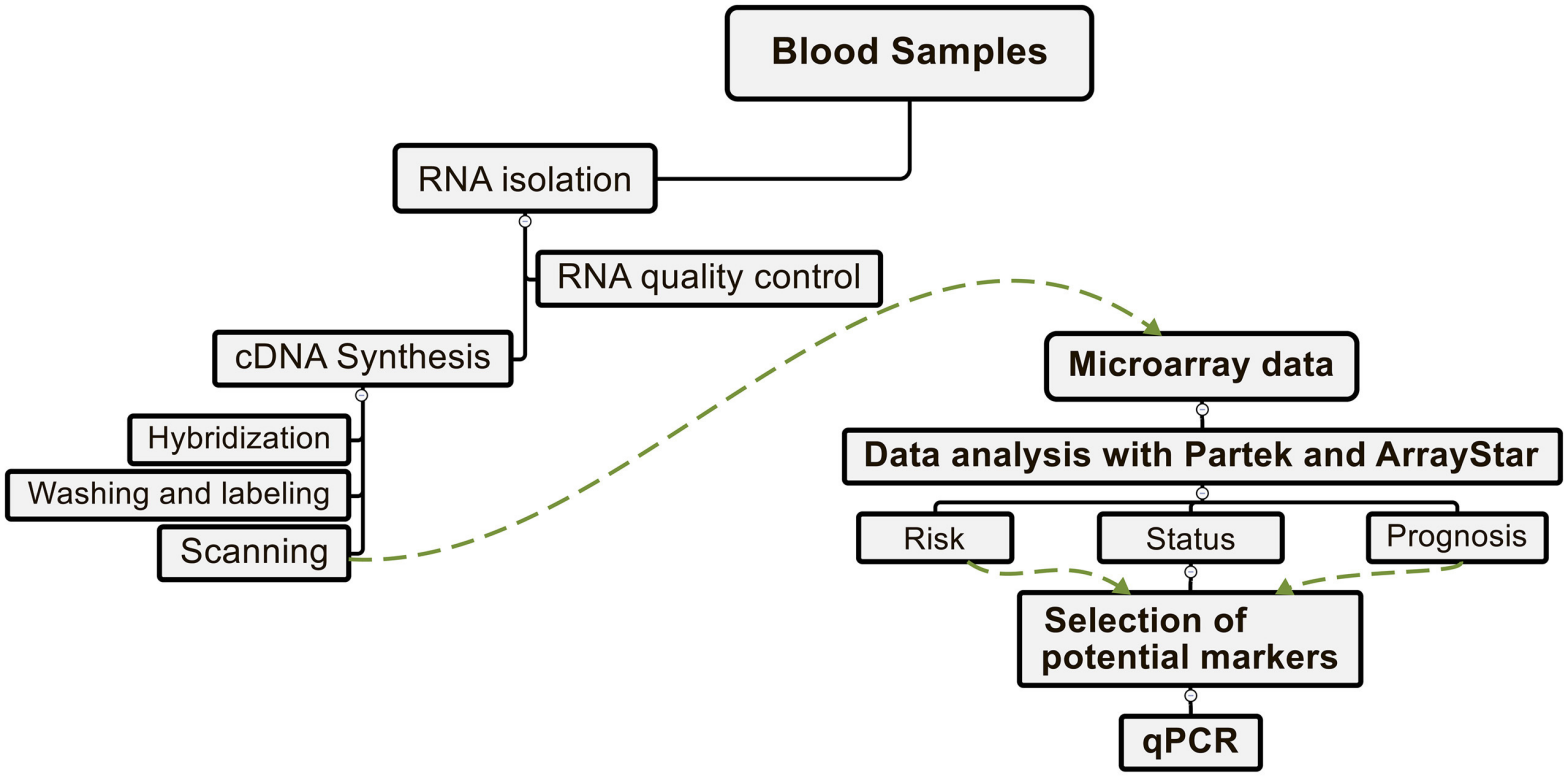
Workflow scheme. From the blood samples, the total RNA was first isolated, subjected to a quality control, and converted to cDNA. Subsequently, after hybridization, labeling, and scanning of the microarray's chips were done. Data analysis in relation to the status of the disease, the risk group and the prognosis of the patients performed with two programs Partek® and ArrayStar®. From the differentially expressed genes the potential markers and verified their expression with a qPCR.

Patients selection was based on the following inclusion criteria: the patient was diagnosed with urothelial carcinoma of the urinary bladder (cystoscopic and histological evidence of Bladder Cancer). Recurrence-negative patients during monitoring have been defined as showing no cystoscopic or histological evidence of bladder cancer. Recurrence-positive patients during monitoring after treatment for *de novo* bladder cancer are identified as showing cystoscopic and histological evidence of bladder cancer. New patients were patients with bladder cancer without a history of UCB. Healthy volunteers had no previous history of bladder cancer or any other cancer (Table 1). The average age of positive cancer patients was 75.4 years and the average age of recurrence-negative patients was 70.5 years. The average age of new bladder cancer patients was 76.7 years and the average age of healthy volunteers was 68 years. Plasma from blood samples was collected from the same cohort of patients except 4 patients and one healthy volunteer were missed. Plasma was obtained using BD Vacutainer® Plasma Tubes (Heparin). Tubes containing 8–10 mL of blood were mixed for 30 min at RT and centrifuged at 1,600 g for 10 min at RT. Plasma was added to a fresh tube and centrifuged again at 1400 g for 10 min at RT. Plasma was then transferred to cryo-vials and stored at −80°C. Whole blood was collected using BD PAXgene™ Blood RNA Tubes. After the patient was bled the PAXgene™ Blood RNA Tubes were incubated at RT for 2 h and then chilled on ice for 10 min. The tubes were then incubated at −20°C overnight and then transferred to a storage box at −80°C. The workflow of the current microarray study from blood samples is illustrated in Figure 1. For EN2 detection using the lateral flow test, 62 urine samples from positive patients with cystoscopy and histological evidence of bladder cancer were used. This group in second analysis was grouped to different stages (Ta = 35 samples, T1 = 7, and T2-T3 = 9) and grades (36–38) (G1 = 11, G2 = 33, and G3 = 17) to evaluate the clinical sensitivity of the rapid test by tumor stage and grade. It was not possible to determine a stage (I) or a grade (II) for only one specimen. Forty-six negative samples were collected from healthy volunteers without any previous history and symptoms of bladder cancer or any other cancer. All urine samples were stored at −20°C.

**Table 1:** Participant and specimen characteristics summary for microarray and plasma.

		**No of TCC patient (transitional cell (urothelial) carcinoma)**						**Muscle invasive**
**Patients**	**No of patients**	**Total No of TCC patient**	**G 1 pTa**	**G 2** **pTa**	**G 2** **pT1**	**G3 pTa**	**G3 T1**	**G3 T2a**
**Risk group**			**Low risk**	**Intermediate risk**	**Intermediate risk**	**High risk**	**High risk**	**High risk**
Recurrence-positive	17	15	4	9	1	0	1	2
Recurrence-negative	18	0	0	0	0	0	0	0
New Bladder Cancer patients	11	9	1	4	3	0	1	2
Health volunteers	29	0	0	0	0	0	0	0

### RNA and Array Processing

Isolation of total RNA from clinical whole blood samples was performed according to the protocol of the manufacturer using the PAXgene® Blood RNA-Kit (PreAnalytiX). The total RNA was quantified by a Qubit™ Assay (Invitrogen) based on the manufacturer's standard protocols. The RNA integrity was determined by agarose gel electrophoresis. Using the manufacturer's protocol, the RNA samples were processed by the GeneChip® WT Plus Reagent Kit (Affymetrix). In summary, the total RNA firstly transcribed to double stranded cDNA, then transcribed to cRNA. The cRNA was then synthesized into single stranded cDNA, and then fragmented and biotinylated. Finally, the biotinylated single stranded cDNA has been hybridized onto the whole transcript Affymetrix® Human Gene 2.1 ST arrays, which cover a total of 40.716 annotated transcripts. The strips were then labeled using a streptavidin phycoerythrin conjugate, washed and scanned using the GeneAtlas® System (Affymetrix). Using the Command Console™ software (Affymetrix), the probe cell intensity data (CEL) files were created.

### Gene and Exon Expression Analysis

For the gene and exon expression analysis, the CEL files are imported into Partek Genomics Suite version 6.6 (Partek Inc., St. Louis, MO, USA) and normalized using the Robust Multi-array Average (RMA) settings. A batch effects removal was performed to minimize the influence of external factors on the data using the tool in Partek software. The lists of differentially expressed genes were created using analysis of variance (ANOVA) with an fdr (false discovery rate) corrected *p* < 0.05 and a fold change >1.5 (39–42). The QC metrics table and QC graphical report was used to assess of the quality the experiments. The average linkage hierarchical clustering was conducted using spearman's correlation as a similarity matrix. Using the Partek Gene Ontology (GO) Enrichment tool, differentially expressed genes were grouped into functional categories ranked according to their *p*-values. Additionally, the differentially expressed genes were plotted in a volcano plot. The differentially expressed genes filter according to their fold-change were used for further functions analysis using Pathway analysis utilizing KEGG database (Partek® Pathway™). The significantly enriched pathways (using a Fisher's exact test) were determined and visualized by Partek Pathway. Enrichment Score (Fisher's Exact test) was used to sort the top enriched pathways out of differentially expressed genes). In the second analysis the CEL file were imported into DNASTAR® ArrayStar® (version 12.0) software (DNASTAR. Madison, WI) using again the RMA normalization. The lists of differentially expressed genes were generated using a Student's *t*-Test with a confidence interval >95% and a fold chance >1.5. Differentially expressed genes were grouped into their functional categories ranked according to their *p*-values using the Gene Ontology (GO) Enrichment tool. Unannotated and duplicate probe sets were removed from the lists.

### Quantitative Real-Time PCR (qPCR)

Expression of selected genes was verified performing a quantitative PCR (qPCR) using the housekeeping gene GAPDH as reference. The primers used are described in Table S1. The analysis was performed in 20 μL containing each 10 μL 2x FastStart Essential DNA Master Mix (Roche), 2.4 μL H2O (PCR grade), 1.3 μL from 8 μM of the respective forward and reverse primers and 5 μL from 5 ng/μL template DNA. As template DNA the single strand cDNA generated for the microarrays was used. No template was included in the negative control. The following steps were included in the PCR Protocol: denaturation at 95°C followed by 9 touch-down cycles with the annealing temperature decreasing 1°C per cycle from 61° to 53°C. Then 31 cycles followed with 20 s at 95°C, 20 s at 53°C, and 20 s at 72°C. Finally, the DNA was denatured at 95°C for 20 s then at 55°C for 60 s and at 97°C for 1 s to generate the melting curves. The qPCR was performed on the Light Cycler® 96 (Roche) and the relative gene expression was determined using the Light Cycler® 96 software (version 1.1). Normalization was performed using the healthy samples as run calibrator. The normalized ratios were calculated as the ratio of the samples (target/reference) divided by the ratio of run calibrator (target/reference) using healthy samples.

### Recombinant S100A12 Synthesis

The target gene was amplified using plasmid pENTR223.1 containing cDNA fragment of human S100A12 (Hölzel Diagnostika, Germany) as template and primers with the 5′-3′-sequences CGCGCGCCATGGTTACAAAACTTGAAGAGCATC and CGCGCGAAGCTTACTCTTTGTGGGTGTGGTAA, respectively. The ends of the PCR product were cleaved using FastDigest restriction endonucleases *NcoI* and *HindIII* (Thermo Scientific). By ligation to IPTG-inducible expression vector pLEXWO481 digested before with the same enzymes set the S100A12 reading frame was fused in frame with the N-terminal His-tag. *E. coli* DH5α (NEB) was transformed using ligation mixture. Afterwards plasmids were isolated from positive transformants and confirmed by sequencing. For production of biomass of recombinant *E. coli* DH5α cells we used previously described conditions (43).

### Purification of His-Tagged Recombinant S100A12

All of the following purification steps were performed on ice or at 4°C. A cell pellet of 9.6 g in weight was thawed on ice and suspended in 20 mM Tris-HCl, 100 mM NaCl, pH 8.0 for 0.1 grams of cell pellet per mL. The cells were agitated gently for ~1 h and homogenized (Miccra D-8). Cell lysis was performed by sonication (Bandelin Sonopuls GM 70, equipped with a UW 70 booster horn). Sonication was performed on ice for 5 × 30 sec. min. at 80% amplitude, with breaks between cycles to prevent warming of the mixture. The lysate was centrifuged at 27,000 × g, 8°C for 30 min to remove unlysed cells and insoluble cell debris. The supernatant (cleared lysate) was filtered through a fluted filter and the pellet was discarded. The following Ni-NTA affinity chromatography and gel filtration was performed with an Äkta prime plus chromatography system (GE Healthcare). The cleared lysate was applied on a column (HiScale 16, GE Healthcare) containing 5 mL Ni-NTA superflow resin that had been pre-equilibrated in 20 mM Tris-HCl, 100 mM NaCl, pH 8.0. After washing the column with 5 column volumes of 20 mM Tris-HCl, 100 mM NaCl, pH 8.0, a linear imidazole gradient from 0 to 1.0 M was applied over 20 column volumes. 2.5-mL fractions were collected over the gradient. The recombinant S100A12 protein was eluted with approximately 250 mM imidazole. Fractions containing S100A12 with a purity of 95% or higher were pooled. Buffer exchange against 10 mM NH_4_HCO_3_, pH 8.0 was performed using a prepacked desalting HiScale 16 column, filled with 40 mL Sephadex G-25 Medium (GE Healthcare). Protein-containing fractions were pooled and filtered through a 0.2 μM PES filter (Millex-GP, Merck Millipore).

### Immunohistochemistry

The tissue array (Biomax BL802a), was subjected to deparaffinization in a series of alcohols and antigen retrieval in boiling in 0.01 M citrate buffer. Slides were blocked in normal horse serum (Vector Laboratories, UK) and Avidin/Biotin blocking kit (Vector Laboratories, UK). Sections were incubated with 1:1,000 S100A12 primary antibody (Sigma HPA002881) or PBS/0.1% BSA (negative control), before adding universal secondary antibody (Vector Laboratories, UK). ABC reagent (Vector Laboratories, UK) was dropped onto sections, followed by DAB substrate solution (Vector Laboratories, UK). Sections were counterstained with haematoxylin (Vector Laboratories, UK), before being dehydrated in a series of alcohols, cover slipped with Vector mounting media (Vector Laboratories, UK), and visualized by light microscopy.

### S100A12 Quantitation in Plasma Samples Using Biolayer Interferometry (BLI)

BLI experiments were conducted using an Octet QKe Instrument and high precision streptavidin biosensor (SAX), manufactured by ForteBio (Menlo Park, CA, USA). By measuring the light interference on the fiber optic sensor surface, and this is directly proportional to the thickness of the surface-bound molecules. Antibodies against S100A12 are chemically attached to the sensor surface using biotin-streptavidin interactions. Binding of S100A12 in the diluted plasma to the tethered antibodies results in surface thickening, which is monitored in real time. Purified rabbit anti-S100A12 polyclonal IgG were obtained from St. John's Laboratory (London, UK). Anti-S100A12 antibodies were biotinylated using Biotin Protein Labeling Kit (Roche) per the manufacturer's instructions. Sephadex G-25 columns (GE Healthcare) were then used to remove unreacted biotinylation reagent and buffer exchange into PBS. The biotinylated anti-S100A12 was immobilized on SAX biosensors at a single concentration of 10 μg/mL for 40 min (online). A solution of 0.1% BSA in PBS was used as blocking agent for 5 min to reduce the impact of non-specific binding to the surface of the sensor the sensors. The regeneration of the sensors was performed with 10 mM glycine buffer (pH = 2.2). All experiments were performed at 30°C with an agitation set at 1,000 rpm using solid black 96-well plates (Greiner Bio-One) with 10 min assay time (read time window) of dipping the prepared sensors in each well. The final volume for all the solutions was 200 μL/well. Different concentrations of recombinant S100A12 (from 1,857 ng/mL to 0 μg/mL) spiked in 1:12 diluted healthy human plasma to stablish the standard curve. The cohort plasma samples were all 1:12 diluted using PBS, pH 7.4 before applying in the assay plate. Data were analyzed with the Octet System Data Analysis software v7.1 (ForteBio, Menlo Park, CA, USA), and the S100A12 concentration in plasma samples was obtained using calibration curve which set up using the BLI response (binding rate) to the spiked rS100A12 concentration. Statistical analysis was performed using the GraphPad prism package. Unpaired *t*-test with Welch's correction was used to test the significance of differences between mean S100A12 concentrations in different patient groups. Receiver operator characteristics (ROC) curves were generated for the S100A12 and the unpaired *t*-test was used to test the area under the curve for significance. A combinatorial analysis of both S100A12 and EN2 as combi-biomarker and receiver operating characteristic (ROC) curve was carried out to using the CombiROC method (http://CombiROC.eu) (44).

### Lateral Flow Prototype Development to Detect EN2 in Urine

The EN2 detection test is a membrane-based test for the qualitative detection of EN2 protein in urine through visual interpretation of color development in the test device. The test is based on the principle of Competitive Enzyme Immune Assay with a single test strip contained within a test cassette. This test strip consists of a proprietary EN2-binding IgG antibody (LIONEX GmbH) coupled to a colored conjugate, and a membrane with one test line and one control line. The test line contains EN2 recombinant protein (LIONEX GmbH), the control line consists of an antibody-binding protein (Rabbit anti mouse IgG, Thermo Scientific Fischer). The urine sample (60 μL) is pipetted into the sample well (S) followed by the diluent buffer (60 μL) 1 min later, the diluted sample passes through the conjugate and the EN2 protein in the sample binds to the conjugate. The EN2-conjugate complex migrates due to the capillary action to the site of the membrane where the recombinant EN2 protein is immobilized (test line) and the competition will take place between the sample EN2 and the coated EN2 on the membrane to the colored particle-labeled specific EN2 antibodies. If EN2 is absent or at a low concentration in the sample the labeled anti-EN2 attaches to the coated EN2 on the membrane and color intensity increases. If a high level of EN2 is in the sample, the EN2 is captured by the labeled anti-EN2 and the complex migrates through the membrane without attaching to the EN2 already coated on the test-region. In consequence the color of the test line appears weaker or no test line appears. The remaining complex migrates further across the membrane to the control zone (“C”). Again, a colored line appears, indicating that the test was performed correctly. After 15 min the intensity in the test line is compared to the Reference Scale card (Figure S1). For a positive result two colored lines appear in “T” and “C” or only one colored line appears in the control zone “C.” The test line “T” can be absent or appears weak. The test will be considered as a negative result when two colored lines appear in “T” and “C”; the test line “T” appears strong. The test result is considered invalid if only the test line appears. The control line plays the role of an internal positive control for the lateral flow test and indicates successful test flow.

## Results

### Gene Expression Analysis of UCB Patients

**Figure 2:**
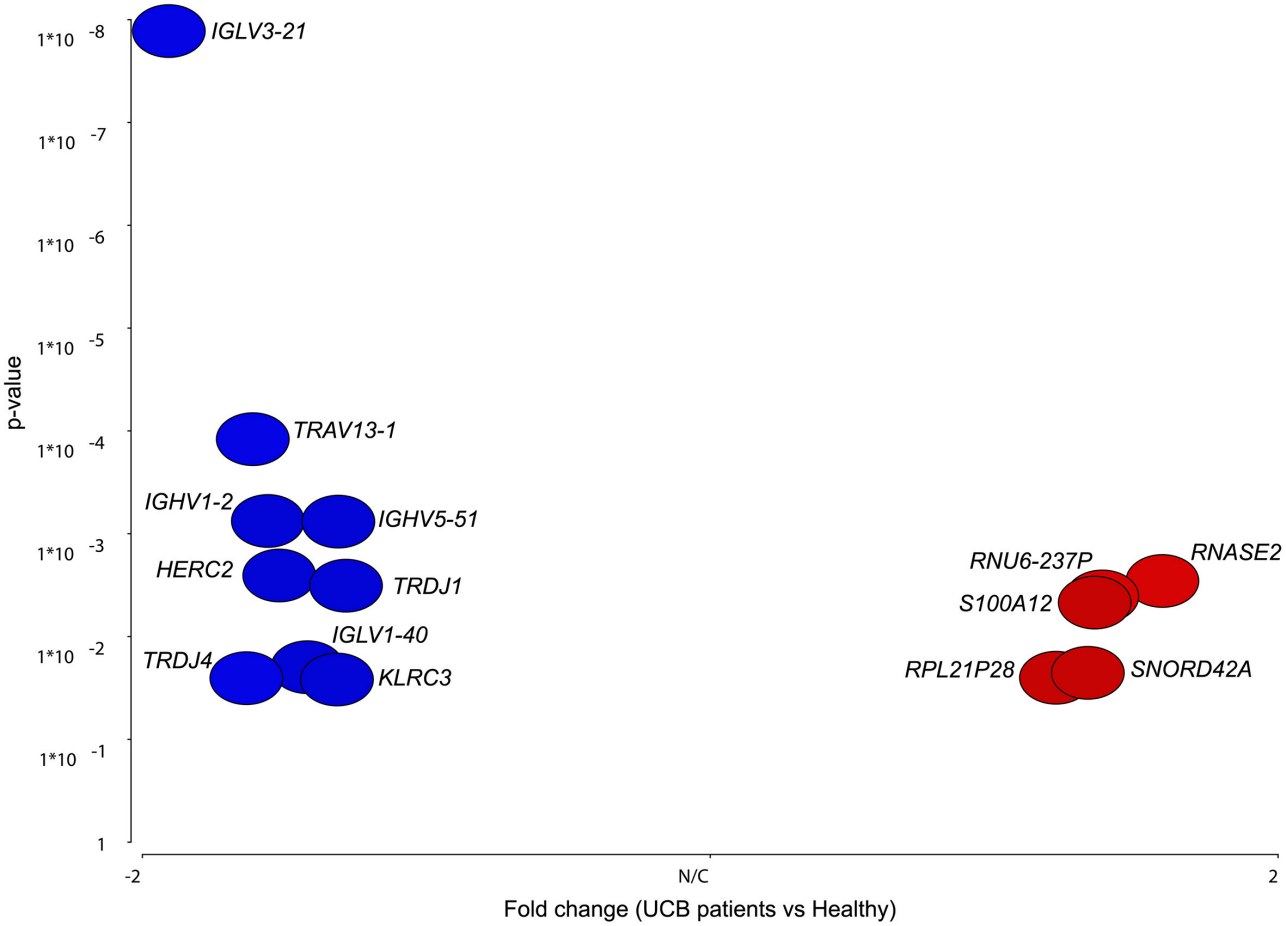
Volcano plot of differentially expressed genes in UCB patients against probability. The figure shows data points of only differentially expressed genes lying above the fold change >1.5 and fdr-corrected *p* <0.05. Points to the right (red) represent candidates that were upregulated in UCB, while points to the left (blue) were downregulated.

We carried out blood sample gene expression profiling in UCB cancer patient groups and/or in comparison to the healthy samples (Table 1). Comparing the expression profiles revealed a set of 14 differentially regulated genes. Of these, 5 were upregulated in bladder cancer while 9 were downregulated (Figure 2). The significance of regulated genes was set based on an fdr-corrected *p*- <0.05 and a fold change >1.5. This criterion was chosen to allow differentially expressed genes to simultaneously meet both fdr-controlled p-value and fold-change requirements even with arbitrarily small fold-changes (45–47). The most significantly upregulated genes included *RNASE2, RNU6-237P*, and *S100A12*, while the most significantly downregulated genes comprised *IGLV3-21, IGHV1-2*, and *TRAV13-1*.

**Figure 3:**
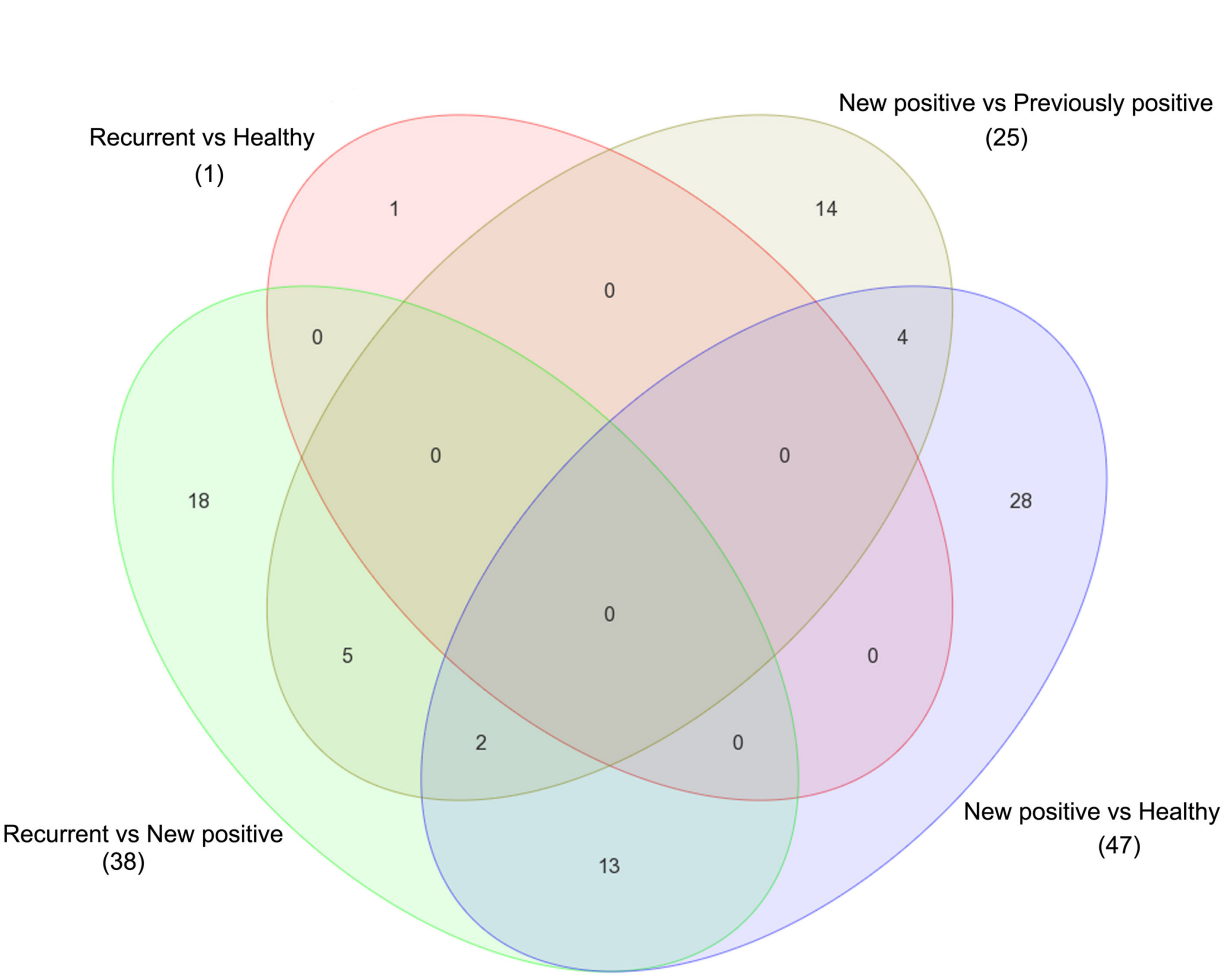
Venn diagram shows the comparison and overlapping of regulated genes in different comparison of UCB according to their prognostic's groups. Significance was selected based on a fold change >1.5 with fdr-corrected *p* <0.05.

Next, the UCB cohort patient samples were divided into different groups according to the prognosis of the patients. New UCB cases were labeled “new positive,” in case of a recurrence of the disease within 5 years after treatment the samples were assigned to the group “recurrent” and patients with a bladder cancer history but negative at the time the sample was taken were placed in the “previously positive” group. The analysis of variance (ANOVA) was carried out between these groups and revealed a total of 127 differentially regulated genes (Figure 3). The comparison between the new positive samples and the negative control samples accounted for most of these genes showing 9 upregulated genes among the new positive patients and 38 downregulated genes. In total the comparison between the recurrent and the new positive samples revealed 38 differentially regulated genes. As shown in Figure 3, 18 of these genes were exclusively found in this comparison as possible prognostic markers to distinguish recurrent cases from new UCB cases (Table S2).

To identify a pattern of expression associated with UCB recurrence, we noticed that *PTGDR, KLRF1* and *MT-TC* genes were found to be significantly downregulated in new positive samples compared to negative and recurrent samples, while *RNU6-135P* was upregulated in new positive samples compared to previously positive and recurrent samples (Table 2). To further investigate the effects of risks groups in UCB, a model was built using healthy, previously positive patients and dividing samples into risk groups according to stage and grade of the disease as defined previously (48) (Table 1). ANOVA analysis carried out between these groups revealed no differentially regulated genes in the comparison between the low risk and negative samples. When comparing high risk and negative controls only the JUP gene was found to be significantly upregulated (fdr-corrected *p* < 0.05; fold change >1.5) (Table S3), whilst a comparison of the intermediate risk group and negative controls revealed a total of 20 differentially regulated genes, 4 of which were upregulated while 16 were downregulated in the intermediate risk group. The most significantly upregulated genes were *RNU6-707P* and *S100A12*. *TRAJ29* and *TRAJ17* were the most significantly downregulated genes (Table S3).

**Table 2:** Selected potential prognostics biomarkers.

**Gene**	**Fold change**		
	**New positive vs. Healthy**	**Recurrent vs. New positive**	**New positive vs. Previously positive**
*KLRF1* (Killer cell lectin-like receptor subfamily F member 1)	−1.87	1.97	−1.57
*PTGDR* (Prostaglandin D2 receptor)	−1.71	1.73	–
*MT-TC* (Mitochondrially encoded tRNA cysteine)	−1.91	2.04	−1.80
*RNU6-135P* (RNA, U6 small nuclear 135, pseudogene)	–	−2.65	1.98

Significantly enriched pathways are shown in Table 3 from the Partek pathway analysis of UCB patients vs. healthy group sorted by the Enrichment Score (Fisher's exact test). This analysis showed that the genes significantly affected by bladder cancer are involved in the antigen processing and presentation, natural killer cell mediated cytotoxicity, and the ubiquitin mediated proteolysis pathways. By analyzing different combinations of the expression data in the prognostic and risk groups, the comparisons showed regulation in several pathways. It was noticeable, however, that the natural killer cell mediated cytotoxicity pathway repeatedly appeared in comparisons of different patient groups against the healthy and within the prognostic/risk groups (Table 4).

**Table 3:** The selected top pathways.

**Pathway name**	**Database**	**Enrichment score**	**Enrichment *p*-value**
Antigen processing and presentation	kegg	3.88856	0.0204748
Natural killer cell mediated cytotoxicity	kegg	3.29709	0.0369907
Ubiquitin mediated proteolysis	kegg	3.22929	0.0395856

*The analysis done using Partek pathway and the data sorted by the Enrichment Score using Fisher's Exact test*.

**Table 4:** Overview of genes differentially regulated in pathway analyses connected to natural killer cells.

**Comparison**	**Pathway**	**Gene**	**Up or down regulated**
Patient vs. healthy	Antigen processing and presentation	*KIR* (Killer-cell immunoglobulin-like receptor)	Down
	Natural killer cell mediated cytotoxicity	*NKG2C/E* (killer cell lectin-like receptor subfamily C)	Down
Intermediate risk vs. healthy	Antigen processing and presentation	*KIR* (Killer-cell immunoglobulin-like receptor)	Down
	Natural killer cell mediated cytotoxicity	*NKG2C/E* (killer cell lectin-like receptor subfamily C)	Down
New positive vs. healthy	Antigen processing and presentation	*CD8* (cluster of differentiation 8)	Down
	Antigen processing and presentation	*KIR* (Killer-cell immunoglobulin-like receptor)	Down
	Natural killer cell mediated cytotoxicity	*NKG2C/E* (killer cell lectin-like receptor subfamily C)	Down
	Natural killer cell mediated cytotoxicity	*NKG2D* (killer cell lectin-like receptor subfamily K)	Down
	Natural killer cell mediated cytotoxicity	*NKG2DL* (killer cell lectin-like receptor subfamily K ligand)	Down
	Natural killer cell mediated cytotoxicity	*Perforin*	Down
New positive vs. previously positive	Antigen processing and presentation	*KIR* (Killer-cell immunoglobulin-like receptor)	Down
	Antigen processing and presentation	*CD8* (cluster of differentiation 8)	Down
Recurrent vs. new positive	Natural killer cell mediated cytotoxicity	*Perforin*	Up

*A pathway analysis was conducted with genes identified as differentially regulated by Partek*.

### *S100A12* Is UCB Relevant and Its Expression Is Elevated in High-Risk Patients

**Figure 4:**
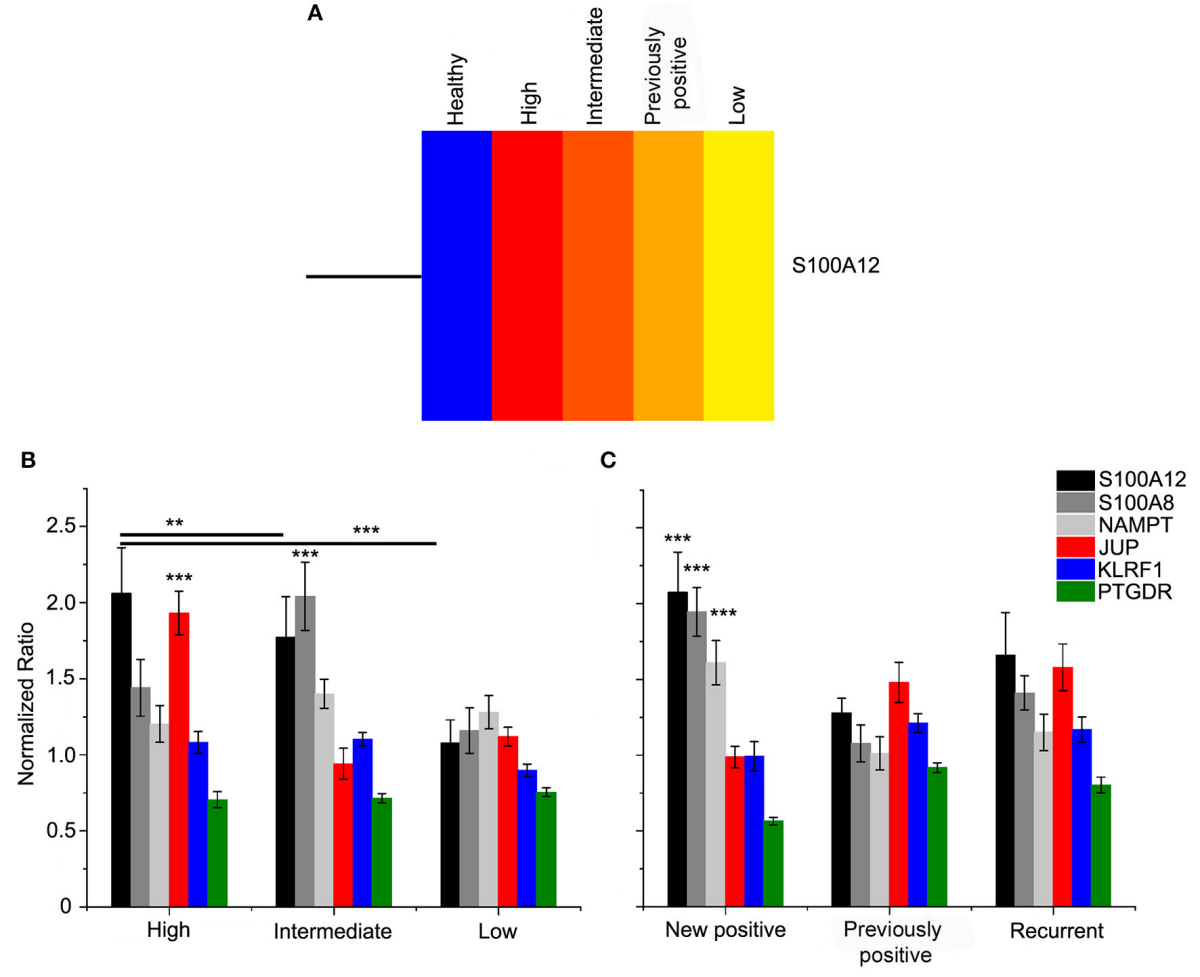
**(A)** Heat map of S100A12 as most significantly regulated gene in risk groups. The gene and samples are clustered using Centroid linkage and Euclidean distance metric by ArrayStar software. The diagram row represents the S100A12 gene and the risk groups are presented in the columns. Color saturation reflects the differences in gene expression between tumor samples; red is higher than control group expression (blue). The intensity of color indicates the degree of gene expression from red (high expression fold) to yellow (low fold expression). qPCR validation of expression was done for selected genes. Normalized ratio ± ratio error determined for cDNA pools of study risk **(B)** and prognostic groups **(C)** by calculation of the ratio of the samples (target/reference) divided by the ratio of run calibrator (target/reference) (healthy samples). Quantitative PCR was performed for S100A12 (black), S100A8 (gray), NAMPT (light gray), JUP (red), KLRF1 (blue), and PTGDR (green). New positive, New UCB cases; recurrent, Cases with recurrence of the disease within 5 years after treatment; previously positive, Cases with UCB history but negative at the time the sample. ***p ≤ 0.0001 and **p ≤ 0.01.

*S100A12* was the only gene which was found to be differentially regulated in several analyses. This gene was significantly upregulated in the comparisons between patients and healthy volunteers as well as between intermediate risk patients and healthy volunteers. In both cases a fold change of 1.6 was associated to this gene. In order to detect potential biomarkers, these results were checked by analyzing again the microarray raw data using DNAStar ArrayStar software. A student's *t*-test was applied with a confidence interval of 95% and a fold change >1.5. We identified a total of 141 differentially regulated genes, all of which were upregulated in UCB patients (Table S4). The top GO functions for disease effects were heavily weighted toward immune system process, regulation, inflammatory response, cellular homeostasis, and cell cycle genes. The list of the cellular homeostasis and cell cycle genes out of GO enrichment clustering was analyzed using ArrayStar, to gain insight into their biological relevance by supervised hierarchical clustering (Figures S2, S3). This analysis also showed a significant upregulation of *S100A12* in bladder cancer patients compared to healthy volunteers. In addition, the analysis of the different risk groups displayed an increasing expression pattern of *S100A12* among these groups (Figure 4A). The most significant upregulation was found among the high-risk patients with a fold change of 3.15. Among the intermediate risk patients, the fold change went down to 2.63 and dropped further to only 1.85 among the low risk patients. Interestingly, in the previously positive group *S100A12* also was found to be upregulated among prognosis group and higher than what seen in low risk individuals (2.15-fold) (Figure 4A). Moreover, in the ArrayStar analysis two genes from the S100 family were found to be differentially regulated in UCB patients compared to healthy volunteers. *S100A8* and *S100A9* were significantly upregulated with a fold change of 1.92 and 1.56, respectively. Another interesting gene showing a significant upregulation in the ArrayStar analysis, which had not been found by Partek software analysis was *NAMPT* with a fold change of 2.05.

**Figure 5:**
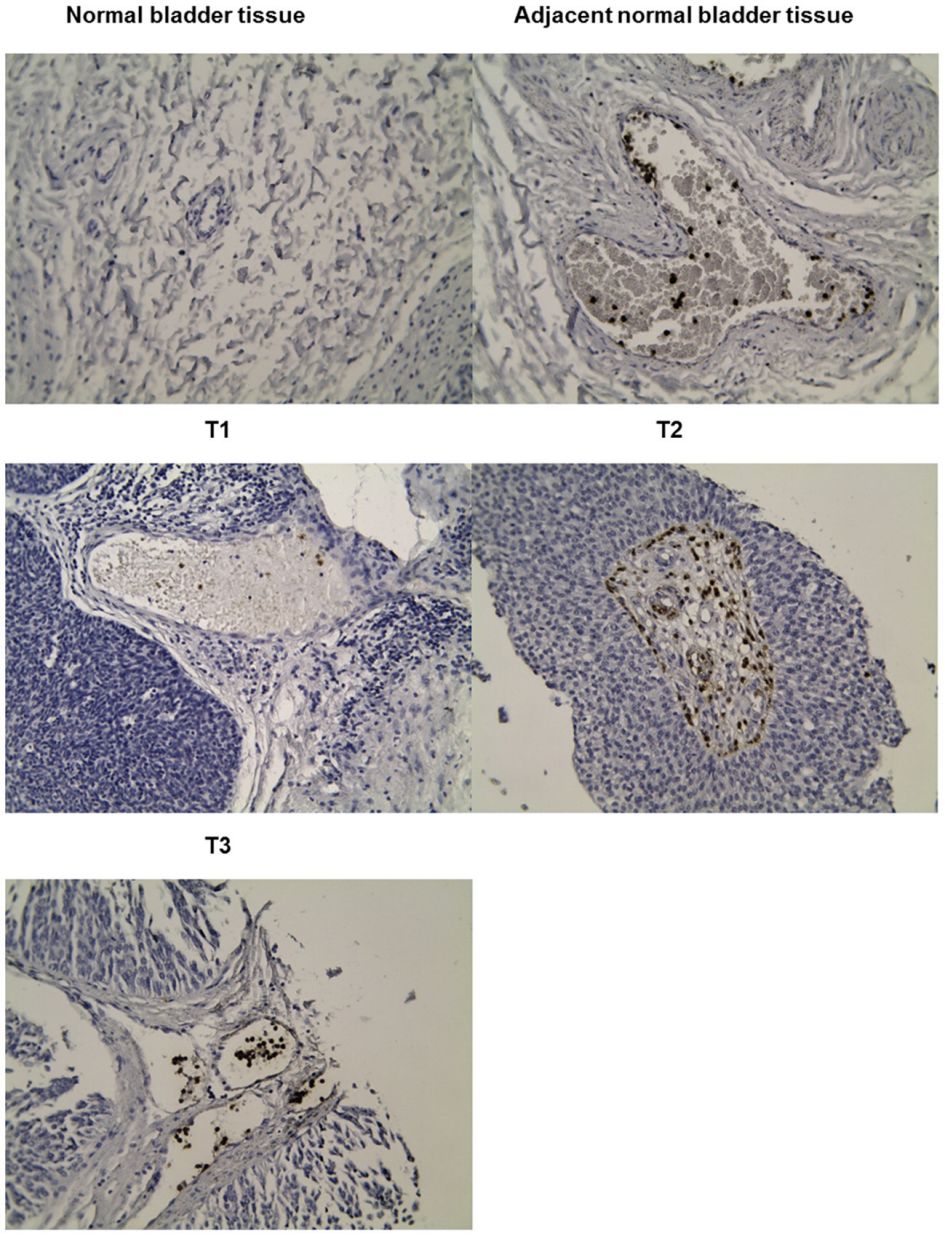
Representative immunohistochemical analysis for tumors of T1, T2, and T3. IHC staining patterns for S100A12 in cancer tissue array tumors stained withanti-S100A12.

To validate the microarray analysis results, the expression of the following selected genes was checked by means of qPCR: *S100A12, S100A8, NAMPT, JUP, KLRF1*, and *PTGDR*. *S100A8* was included in this analysis, although this gene did not show a significant change among all groups in the ArrayStar analysis. However, it appears in the literature together with *S100A12* as a potential biomarker of bladder cancer in human and dogs (30, 32, 35). The real-time qPCR was performed using the housekeeping gene *GAPDH* as reference. The cDNA samples were pooled according to their risk and prognostic groups and the cDNA of each sample was present in equal amount. The analysis of the risk pools resulted in an expression pattern for *S100A12* similar to ArrayStar analysis and it could be confirmed that the expression increases with risk (Figure 4B). *S100A8* was shown to be 2-fold upregulated in intermediate risk patients, in high risk patients the fold change was 1.4 and in the low risk group it was 1.2. Surprisingly, *JUP* showed a significant increase among the high-risk group. Through the prognostic groups, the expression of *S100A12, S100A8*, and *NAMPT* in the new positive patients is significantly higher than that of previously positive and recurrent groups (Figure 4C). In contrast, the overexpression of *KLRF1* and *PTGDR* could not be demonstrated. Considering this profound microarray expression of *S100A12*, we screened a human bladder cancer tissue array using IHC with an antibody to S100A12. The tissue array contained, 1 squamous cell carcinoma, 2 adenocarcinoma, 57 cases of urothelial carcinoma, 10 normal bladder tissue and 10 each of adjacent normal bladder tissue, a single core per case. S100A12 appears to be mostly expressed in cells contained within the stroma and not in the tumor tissue. Necrotic areas showed non-specific staining which was therefore excluded from the analysis. Figure S4 shows the percentages of cells that were positive for S100A12 in each tissue sample group (normal bladder, adjacent normal bladder tissue, T1-3 stage tumors and G1-3 grade tumors). The data shows normal bladder has the lowest expression of S100A12 and that expression rises with stage and grade. It is clear that the highest expression is present in the normal bladder tissue adjacent to the tumor suggesting immune infiltration by leukocytes (Figure 5).

### Plasma S100A12 Concentrations Are Predictive for UCB

**Figure 6:**
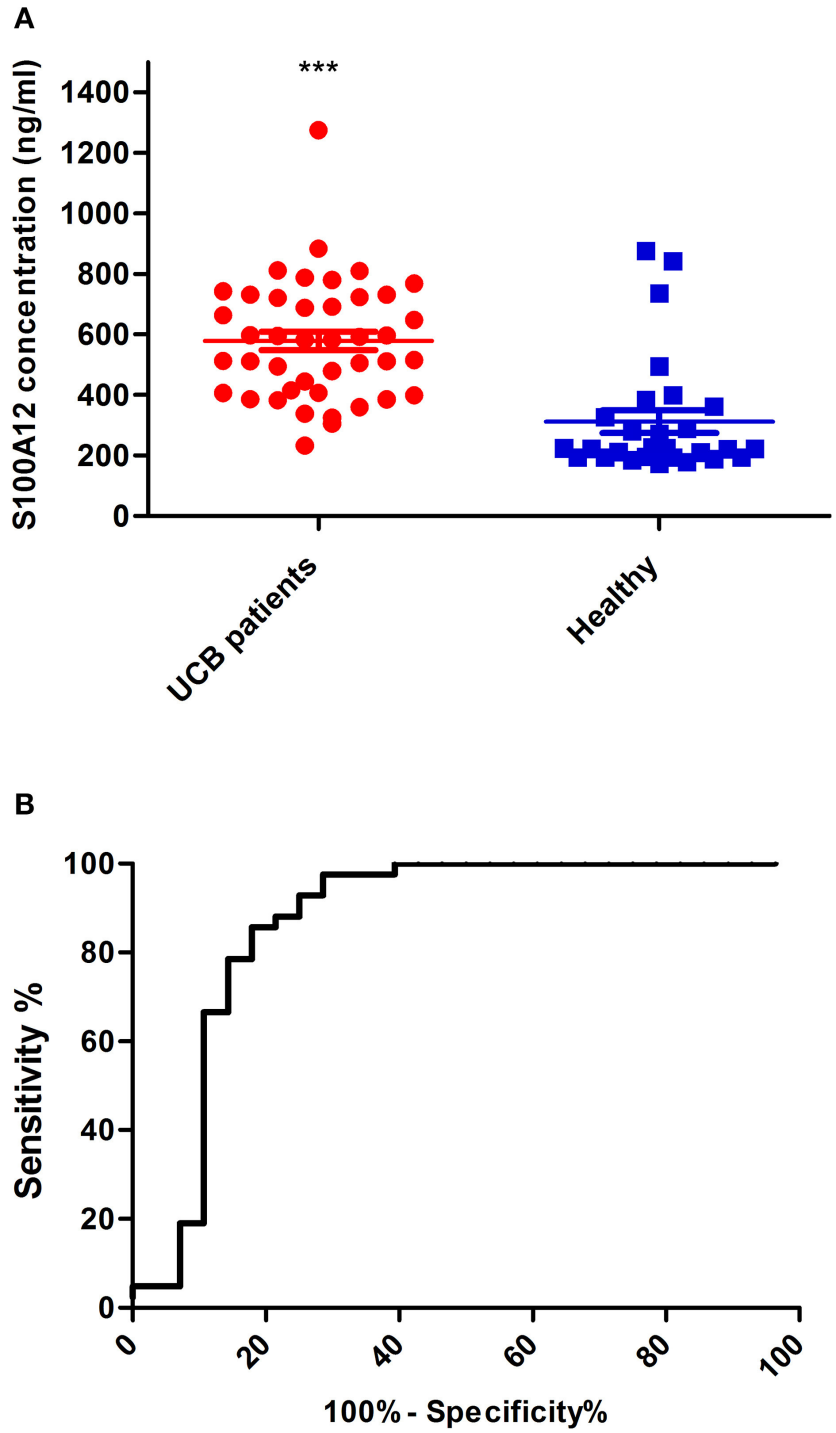
Quantitation of S100A12 in UCB plasma samples by BLI **(A)**. The concentration of S100A12 was measured from plasma samples of each group (UCB patients and Healthy) using the biolayer interferometry (BLI). For each group, the mean value for of S100A12 is shown and standard error of the mean is represented by error bars. **(B)** A ROC analysis of plasma S100A12 concentrations in all UCB patients vs. all healthy in study cohort ***p ≤ 0.0001.

**Figure 7:**
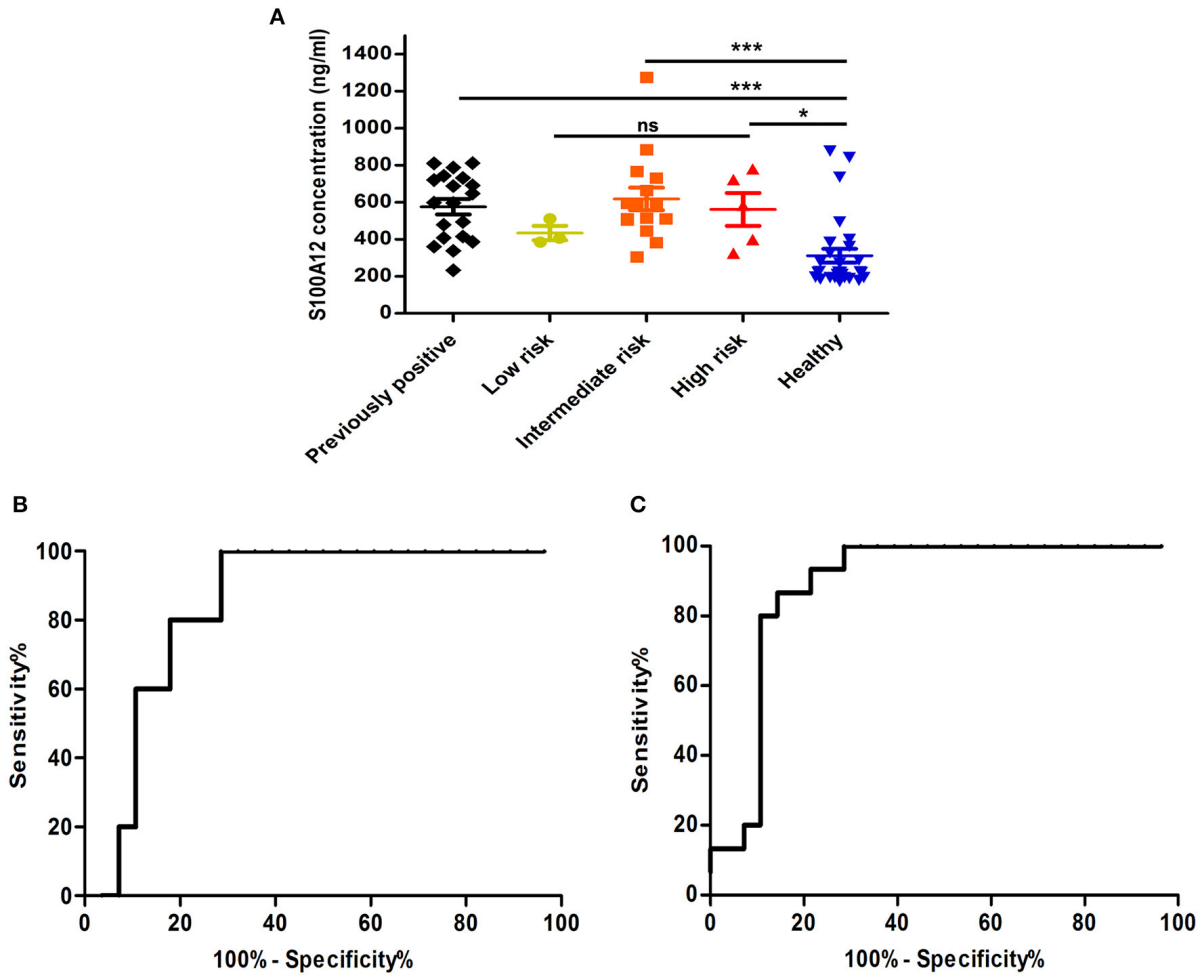
A comparison of S100A12 concentrations in plasma within risk groups. **(A)** Comparison of plasma concentrations of S100A12 in UCB patients divided into groups according to their risk score. The standard error of the mean is represented by error bars. **(B)** A ROC analysis of plasma S100A12 concentrations in high risk UCB patients vs. healthy group. **(C)** A ROC analysis of plasma S100A12 concentrations of intermediate risk UCB patients against study healthy. ns, not significant, ***p ≤ 0.0001 and *p ≤ 0.05.

The presence of S100A12 in plasma would indicate that it could be a potential diagnostic marker, and indeed western blotting revealed that full-length protein S100A12 could be detected in the plasma of UCB patients (data not shown). To quantify the S100A12 concentration in UCB samples, the S100A12 was analyzed in plasma samples using biolayer interferometry (BLI). We assessed plasma S100A12 levels in the same microarray cohort of patients and controls. We found that S100A12 levels in those diagnosed with UCB were significantly higher than in healthy volunteers, *p* < 0.0001. The mean plasma S100A12 concentration in patients with UCB was 579.4 ± 30.58 ng/mL, whilst that for control subjects was 311.9 ± 37.03 ng/mL (Figure 6A). For test performance, the receiver operating characteristic (ROC) area under the curve (AUC) appeared to be 0.869 with standard error 0.05426 and *p* < 0.0001, with a sensitivity of 90.5% and a specificity of 75% (see Figure 6B). The ROC analysis study showed that the ideal cancer-vs.-control plasma concentration threshold of S100A12 was 350 ng/mL to maximize the test's sensitivity and specificity. The 95% confidence interval appeared to be 0.7627–0.9754. Higher risks and grade tumors (grades 2 and 3) were connected to higher mean plasma S100A12 concentrations (618.3 ± 60.59 ng/mL for grade 2 and 562.1 ± 88.46 ng/mL for grade 3) than grade 1 tumors (434.5 ± 38.92 ng/mL) (Figure 7A). Area under the ROC curve for high risk patients against healthy was 0.85 with standard error 0.067, and a 95% confidence interval of 0.7180 to 0.9820 (Figure 7B). Using an unpaired *t*-test the difference in the mean value for high risk vs. healthy was found to be statistically significant (*p* = 0.047). The best cut-off in this comparison was found to be 306.9 ng/mL, which gives a sensitivity of 100% and specificity of 71.43%. The area under the curve rises when comparing intermediate risk group of cancer patients to healthy group (Figure 7C). The area under the ROC curve is 0.888 with standard error 0.052, *p* < 0.001 and a 95% confidence interval of 0.785 to 0.991. The *t*-test showed that difference in the mean value is statistically significant (*p* ≤ 0.0001). The best cut-off in this contrast was found to be 372.7 ng/mL, which gives a sensitivity of 93.3% and specificity of 78.5%.

**Figure 8:**
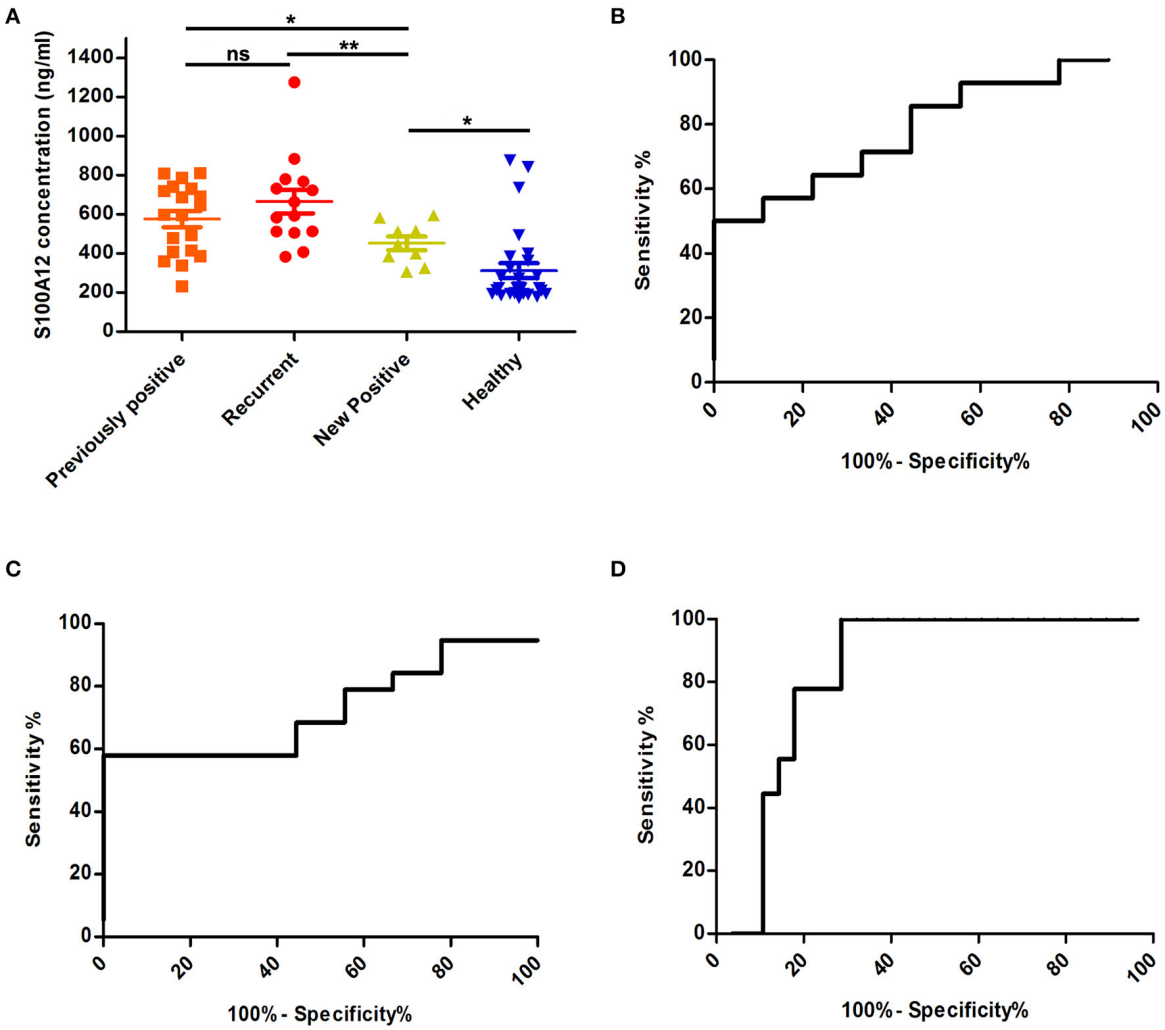
Comparison of S100A12 levels in UCB patient prognostics groups. **(A)** S100A12 concentrations in plasma from UCB patients sorted according to their prognosis. The standard error of the mean is represented by error bars. **(B)** The ROC analysis of plasma S100A12 concentrations of recurrent group against new positive group of patients. The AUC is 0.793 with standard error 0.09, *p* = 0.019 and the 95% confidence interval appeared to be 0.6114–0.9759. The best cut-off in this comparison was found to be 512.5 ng/mL, giving 71.4% sensitivity and 66.67% specificity. **(C)** A ROC analysis of S100A12 concentration of previously positive group (recurrent-negative at sample) compared to new positive group. The AUC is 0.725 with standard error 0.095, *p* = 0.058 and 95% confidence interval between 0.5387 and 0.911. Using the cut-off 462.5 ng/mL the sensitivity is 68.4% and specificity will be 55.56%. **(D)** ROC analysis of S100A12 of new positive UCB patients against all healthy. ns, not significant, **p ≤ 0.01 and *p ≤ 0.05.

To test if S100A12 can also serve as a recurrence and prognostic marker, we performed a ROC analysis in the study prognostics groups. This indicated that the S100A12 concentration could differentiate the UCB-recurrent group and the previously positive group (recurrent-negative at sample) from the new positive UCB patients with an area under the curve of 0.793 and 0.725, respectively (Figures 8A–C). S100A12 also had diagnostic potential for new UCB patients in this analysis, with an area under the curve of 0.833 (Figure 8D), a standard error of 0.065 and p-value of 0.0002. The 95% confidence interval is 0.704–0.9623. Our results showed that best cut-off can be set to be 296.9 ng/mL, which gives a sensitivity of 100% and specificity of 71.43%.

### EN2 Lateral Flow Prototype Validation

In this study, we also assessed the performance of the EN2 rapid test prototype in terms of the sensitivity and specificity of the test. This lateral flow based rapid test is intended as an *in-vitro*-diagnostic test, use of the device is not invasive. All urine samples are coded by an anonymous sample number. One hundred and seven coded urine sample, comprising sixty-two patients and forty-six healthy samples were used for the test prototypes. Each urine sample (60 μL) was applied without any dilution to the test device sample well. Sixty microliters of diluent buffer was applied after 120 s and the test was left for an additional 20 min. Test line intensity was interpreted visually after 15- and 20-min by comparing the test line to the reference card (Figure S1). Criterion for re-examination was that no control line appeared. The results of EN2 rapid tests were compared with cystoscopic and histological evidence of UCB. In the first analysis, the clinical specificity and sensitivity were determined for all positive samples with clinical evidence of bladder cancer vs. all negative samples from healthy volunteers with no history of non-bladder cancer, and active cancers (49–56). The results are summarized in Table 5A. In the second analysis, the clinical sensitivity of the lateral flow test was determined for tumor stage and grade using the positive urine specimens confirmed by means of cystoscopy and histology (62 positive samples). It was not possible to determine a stage or a grade for only one specimen. Stage and grade of the tumor, as well as its detection by the test, are presented in Table 5B. Overall, the sensitivity of the test was 85.48% (95% CI: 74.22–93.14%) at a specificity of 71.74% (95% CI: 56.54–84.01%). This result indicated that the test can potentially distinguish between cancer patients and healthy individuals.

**Table 5A:** EN2 lateral flow test compared by results of clinical diagnosis (cystoscopy and histology method).

**Group**	**Number of specimens**	**Clinical sensitivity in %** **(95% confidence interval)**	**Clinical specificity in %** **(95% confidence interval)**
Patients (cystoscopy positive or/and evidence) (TP/FN)	62 (53/9)	85.48 (74.22–93.14)	71.74 (56.54–84.01)
Healthy (No history or evidence) (TN/FP)	46 (33/13)		

*Exact Clopper-Pearson confidence intervals were used for sensitivity and specificity*.

*TP, true positive; FN, false negative; TN, true negative and FP, false positive*.

**Table 5B:** Sensitivity of EN2 lateral flow by tumor stage and grade.

**Stage of the tumor (I)**	**Number of specimens**	**Clinical sensitivity in %** **(95% confidence interval)**
Ta	35	88.57 (73.26–96.80)
T1	17	94.12 (71.31–99.85)
T2-T3	9	55.56 (21.20–86.30)
**Grade of the tumor (II)**	**Number of specimens**	**Clinical sensitivity in %** **(95% confidence interval)**
1	11	90.91 (58.72–99.77)
2	33	87.88 (71.80–96.60)
3	17	76.47 (50.10–93.19)

## Discussion

Despite the large and growing list of candidate protein markers for UCB (5–11), as yet none have entered routine clinical use. There is no doubt that clinicians need better methods for individual patients' treatments and follow-up regimens selection. It is therefore of paramount importance to recognize novel and validated UCB biomarkers for the detection of disease its recurrence. Using microarrays, different studies focused on UCB global expression profiling (11, 57–61). The main objective of the current study was identifying biomarkers that will predict the likelihood of progression in patients with high grade tumors. By utilizing gene expression profiling, we identified different genes as a signature biomarker for UCB and tumor progression using risk and prognostics grouping. In this study, we used a higher stringency fdr-corrected *p*-value score cut-off of <0.05 combined with batch effects removal to minimize any technical sources of variation (62–64).

Initial comparison was performed on UCB patients using the healthy group samples as control set. This comparison resulted in a set of 14 and 141 differentially regulated genes using Partek and ArrayStar software, respectively. Interestingly, *S100A12* is one of these genes which detected by both software packages to be upregulated in UCB patients (Figure 2). Further grouping of the UCB patients based on their prognosis resulted in a set of 127 differentially regulated genes. Among this multigene expression signature in UCB blood samples, we can identify new potential biomarkers for the prognosis of bladder cancer. *KLRF1* encodes the killer cell lectin-like receptor F member 1, which is expressed on human natural killer (NK) cells and different subsets of T cells (65). It has been shown that a ligand of this receptor (activation-induced C-type lectin, AICL) is produced by hematopoetic as well as non-hematopoetic tumor cells. Blocking of the interaction of AICL and *KLRF1* led to a partial inhibition of NK cell degranulation (65), showing that these receptors play a key role in the killing of tumor cell by NK-cells. A differential regulation of this gene in connection with cancer has not yet been described. Our microarray results however showed that the expression of this gene in new positive patients is considerably lower than in recurrent or previously positive bladder cancer patients. *PTGDR* is also expressed at a significantly lower level in new positive patients than in recurrent and previously positive patients. This gene codes for the prostaglandin D_2_ receptor, which is expressed in different types of cells including immune system cells (NK cells, dendritic cells) as well as cells of central nervous system and smooth muscle cells (66). The receptor is activated by prostaglandin D_2_ which is involved in a variety of different processes like sleep, regulation of body temperature and release of hormones. It also inhibits the aggregation of platelets and promotes relaxation of smooth muscles. It has been shown that the expression of this gene is downregulated in colorectal adenocarcinomas (67) and that there is a correlation between this dysregulation and disease progression as well as a hypermethylation of the gene. Similar hypermethylation patterns have also been demonstrated in the case of bladder cancer (68), although expression of the target gene itself was not been examined. As shown in Table 2 our analysis also revealed other markers in UCB, including *MT-TC* (mitochondrial tRNA cysteine), and *RNU6-135P* (RNA U6 small nuclear 135). *MT-TC* has no known role in cancer, although the expression of tRNA genes in general as well *MT-TC* specifically are substantially upregulated in breast cancer (69). We therefore assumed that downregulation of each of the four genes or at least three of the four biomarkers, would be associated with a higher risk of disease recurrence as proposed for cell cycle regulators (p53, pRB, p21, and p27) (70–72). For searching expression patterns between UCB samples based on risk classification the UCB samples were sorted according to clinical risk score. Our microarray analysis revealed that *JUP* was significantly upregulated in samples of high-risk patients with high grade and stage bladder tumors. This gene encodes plakoglobin (also known as γ-catenin), which has been reported to be involved in the reduction of *in vitro* cell proliferation, invasion and migration (73) as well as the induction of apoptosis (74). Rieger-Christ et al. reported that *JUP* acts as bladder tumor suppressor and that silencing of this gene in late stage UCB is associated with tumor progression (75). It has however also been demonstrated that wild-type and several mutated amino-terminal forms of plakoglobin transformed activity on RK3E epithelial cells (76). Importantly, when we carried out a pathway analysis for our dataset, the natural killer cell mediated cytotoxicity pathway appeared to play a major role in the context of UCB. This is supported by the finding that natural killer cells are essential for bladder cancer therapy with BCG (Bacillus Calmette-Guérin), which is frequently administered to treat superficial tumors (77). In addition, the genes listed in Table 4 have been shown to be important for natural killer cell mediated cytotoxicity. The receptor *NKG2D* for example plays a central role in the recognition of UCB cells by natural killer cells (78). Here we show a downregulation of this receptor. This could be due to an interaction with its ligands, which has been shown previously to downregulate its expression (78). Furthermore, it has been shown that perforin, which was upregulated in this analysis, plays an important role in the lysis of UCB cells by natural killer cells (79).

Characteristics features of the effective biomarkers include cancer-specific expression and tumor release (15). One gene group that has been shown to have these properties recently is the S100 protein family and *S100A12* in particular (21, 24, 29, 32, 34, 35), which is expressed in different type of cancers. In our analysis, *S100A12* was found to be differentially expressed among UCB samples and between risk groups. For further analysis, *S100A12* and *S100A8* are selected based on the results of the UCB expression profile. A clear correlation between the qPCR assays and the microarray data is observed, especially in case of *S100A12* (Figure 4). Both genes were found to be independent and significant prognostic markers in UCB patients. Our results also indicated that, as molecular biomarkers, the products of these genes may be more robust in identifying the high mortality risk group than others with grade 1 disease, which may need to be confirmed with further investigations.

It is generally acknowledged that RNA expression level of a gene does not always reflect the protein expression level, and thus, in order to investigate the eligibility of S100A12 as candidate body fluids biomarker, we decided to measure the concentration of S100A12 in UCB plasma samples and compare it to healthy group samples. Using the 350 ng/mL as cut off, our data showed that S100A12 has a sensitivity of 90.5% and a specificity of 75%. The 100% specificity can be achieved using this assay, with a cut off at 880 ng/mL, the resulting sensitivity, however, is only 4.8%. The maximum sensitivity (100%) is obtained with a cut off at 230.4 ng/mL and this linked with 60% specificity. The cut off value of 350 ng/mL has been selected to provide high sensitivity and specificity. S100A12 could also serve as a prognostic biomarker because our study showed that overexpression of S100A12 protein was associated with recurrence of the disease as well as with high-grade/stage tumors (Figures 7, 8). Unexpectedly, our data also showed that patients with a bladder cancer history, but negative at the sampling “previously positive” group had elevated S100A12 compared to healthy subjects. Similar findings were reported for p53 expression in 692 treated patients with advanced UCB (70) as well as for Ribonucleotide reductase subunit M1 (RRM1) as a prognostic biomarker (80). S100A12 has been shown to be secreted from important inflammatory effector cells such as neutrophils, monocytes, and macrophages (81) and is recognized as having a significant role in inflammation (24, 27, 30–32, 34, 35, 82, 83). A number of studies reported that S100A12 is markedly expressed in several inflammatory disorders such as atherosclerosis, inflammatory bowel disease, Kawasaki disease and coronary artery disease (84). S100B is expressed to differing degrees in normal tissue such as melanocytes, astrocytes, maturing oligodendrocytes, dendritic cells, Langerhans cells, kidney epithelial cells, and certain lymphocyte subpopulations (85). Similarly, S100A8/9 were reported to be markers for UCB recurrence and grade, respectively (33). For UCB our data showed that expression of S100A12 found at the highest level in normal bladder tissue adjacent to the tumor and at much lower levels in tumor itself and normal tissue. S100A12 expression are in line with a previous study in squamous cell carcinoma (86). This could suggest that the S100A12 signal in bladder may be due to an immune infiltration by leukocytes such as neutrophils, monocytes, and macrophages. Further work is needed to be done to define which cell type is involved in this infiltrate. Although our samples are limited and S100A12 mRNA has been reported and linked with UCB (34, 87), to the best of our knowledge, the current study is the first to indicate an increased level of S100A12 protein and mRNA in human UCB and confirm the association between S100A12 and progression of UCB. Hence, we propose the utility of S100A12 at both the mRNA and protein level as a potential marker for UCB detection and prognosis. In addition, the relatively small number of samples had somewhat hampered our data. The current results do not comprise final validation of the clinical uses of S100A12 in UCB detection and prognosis. We have initiated further work using an additional validation cohort to confirm the diagnostic utility of this biomarker.

Additionally, we have previously shown that EN2 is also a potential diagnostic marker in UCB (15). Rapid lateral flow tests, although limited, might be more suitable for use due to their stability, user-friendliness, cost effectiveness, reproducibility, and rapidness (88). Our attempts to develop a prototype lateral flow for qualitative detection of EN2 in human urine were very successful. The sensitivity of the developed test was 85.48% (95% CI: 74.22–93.14%) at a specificity of 71.74% (95% CI: 56.54–84.01%). The test sensitivity varies depending on tumor stage and grade between 55.56 and 94.12% (Table 5B). Thus, the overall sensitivity and the specificity of the EN2 rapid test reaches or surpassed the sensitivity and specificity of many bladder cancer markers and tests on the market (38, 89, 90). These results may be useful for further development of a highly efficient non-invasive and improved diagnostic test. The usefulness of combing S100A12 and EN2 in a single test was assessed using CombiROC tool. Using a cut off 225 ng/mL as an optimal for S100A12, the area under curve (AUC) surprisingly rises to 0.93, and this associated with 92.5% sensitivity and a specificity of 83.1% (Figure S5). This shows that the combination of both biomarkers may equal or exceed the diagnostic performance of other promising UCB markers (5, 6, 8–10, 60, 72, 87).

In summary, in this study we examined the gene expression profile of UCB patients samples and identified several genes with potential diagnostic value by grouping and comparing UCB samples according to their clinical risk and prognostics scores. It is worth noting that these potential markers may be targets for protein and molecular-based clinical diagnosis and/or management of UCB. Importantly, our data revealed a significant increase in the UCB patients in the mRNA and protein expressions of S100A12. We conclude that S100A12 is an independent and significant prognostic marker for UCB patients, which may predict the disease course of UCB patients and facilitate the clinical management of this cancer. We report here also EN2 as diagnostic marker and its performance in prototype rapid lateral flow test assay looks very promising. The prototype performance encourages us to optimize the current design, perhaps adding S100A12 to improve sensitivity and specificity.

## Data Availability Statement

The datasets generated and analyzed in this study are available under the following link in the Gene Expression Omnibus (GEO): https://www.ncbi.nlm.nih.gov/geo/query/acc.cgi?acc=GSE138118.

## Ethics Statement

The studies involving human participants were reviewed and approved by Faculty of Health and Medical Sciences, University of Surrey local ethical committee (Ref. 3/LO/0739). The patients/participants provided their written informed consent to participate in this study.

## Author Contributions

AE, SKl, SKä, WO, MSt, CS, and GS performed the laboratory experiments. AE, SKl, RM, HP, and MSi designed the study and wrote the paper. The final manuscript was reviewed and approved by all authors. All authors participated in data collection and analysis.

### Conflict of Interest

AE, SKä, WO, MSt, and MSi are employed by LIONEX GmbH. SKl was employed during DIPROMON project by LIONEX GmbH. For uses of described biomarkers in this article, AE, SKl, WO, MSt, and MSi have filed a patent application No.: 16203695.8 and patent No.: 1405 under the title: Novel Human Bladder cancer Biomarkers and their Diagnostics use. The remaining authors declare that the research was conducted in the absence of any commercial or financial relationships that could be construed as a potential conflict of interest.
