# Supplementary material for: A Specific Blood Signature Reveals Higher Levels of S100A12: A Potential Bladder Cancer Diagnostic Biomarker Along With Urinary Engrailed-2 Protein Detection
Source: Front Oncol. 2020 Jan 9;9:1484. doi: 10.3389/fonc.2019.01484 (PMC6962349; doi:10.3389/fonc.2019.01484)
Supplement: Supplementary file 1 [file Data_Sheet_1.PDF]

Supplementary Material

1 Supplementary Figures

1.1 Figure S1: Reference card of EN2 lateral flow test.

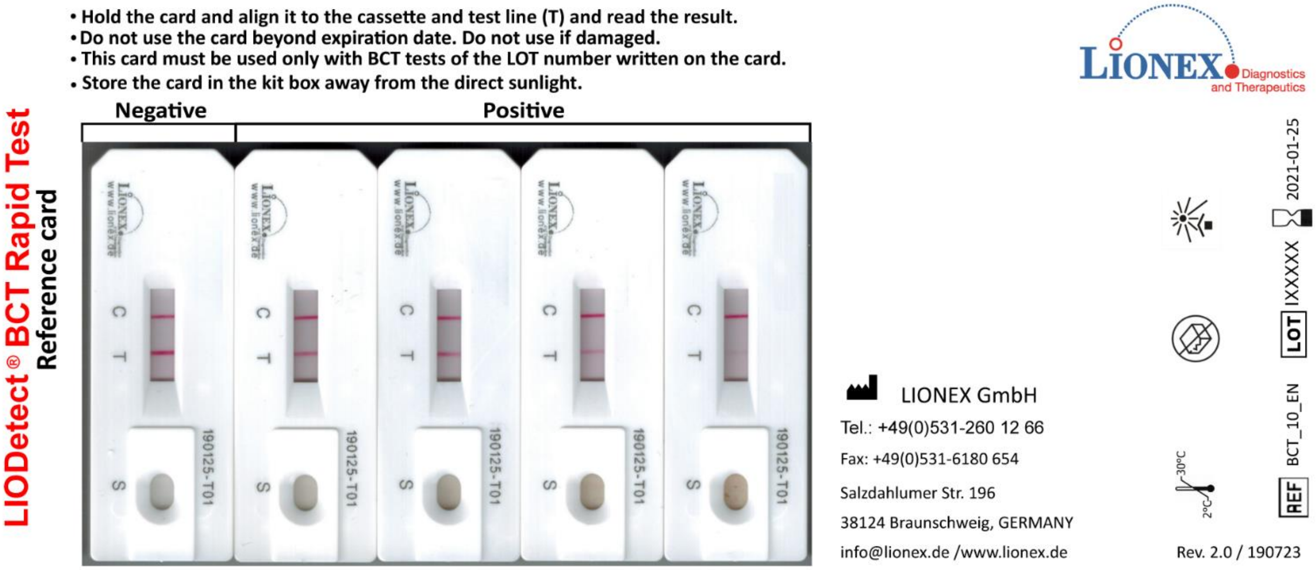

1.2 Figure S2: Hierarchical clustering of significantly gene expression across the list of the cellular homeostasis targets.

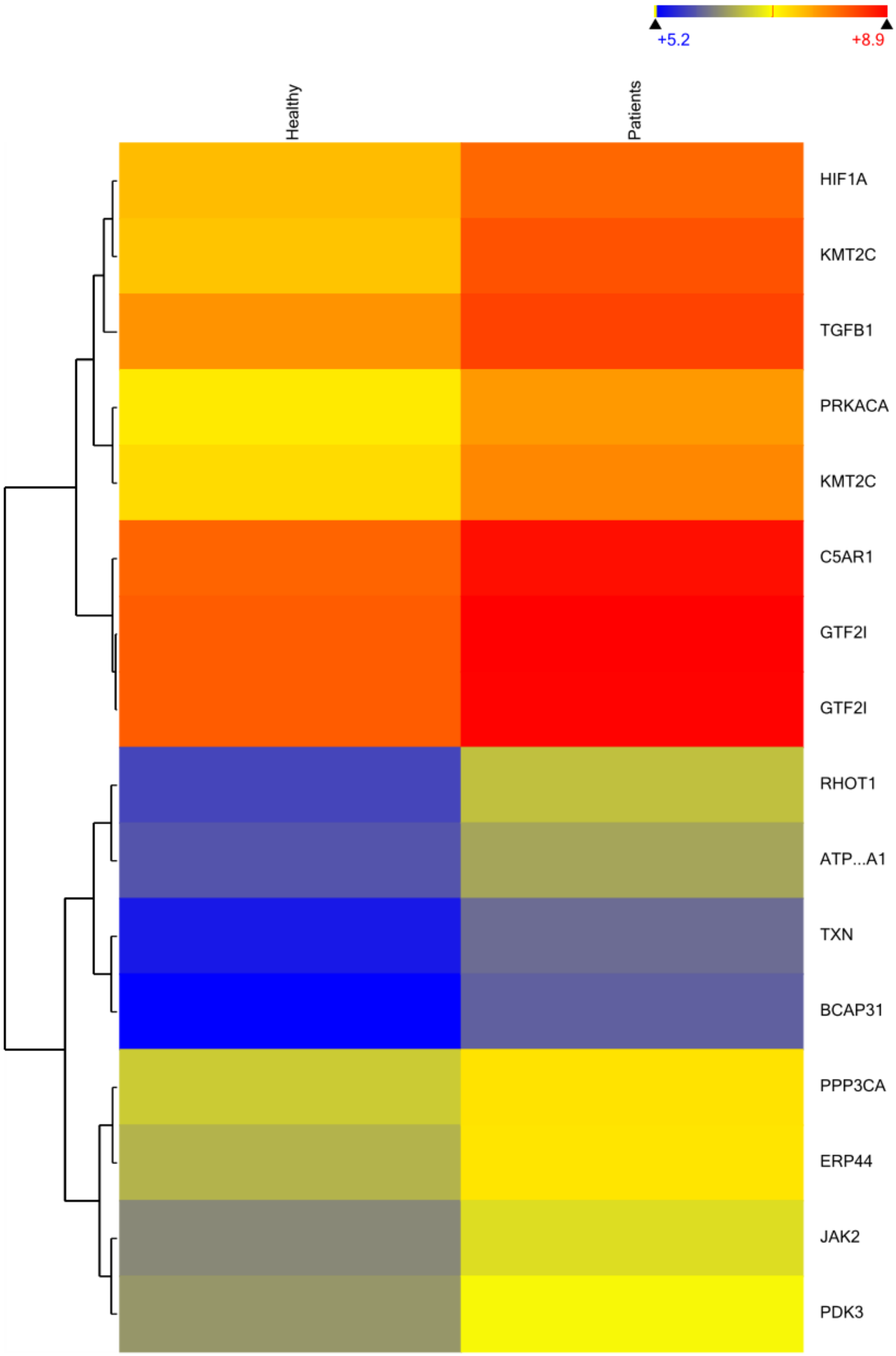

1.3 Figure S3: Hierarchical clustering of significantly gene expression of the list of the cell cycle genes ranked according to their p-values using the Gene Ontology (GO) Enrichment tool.

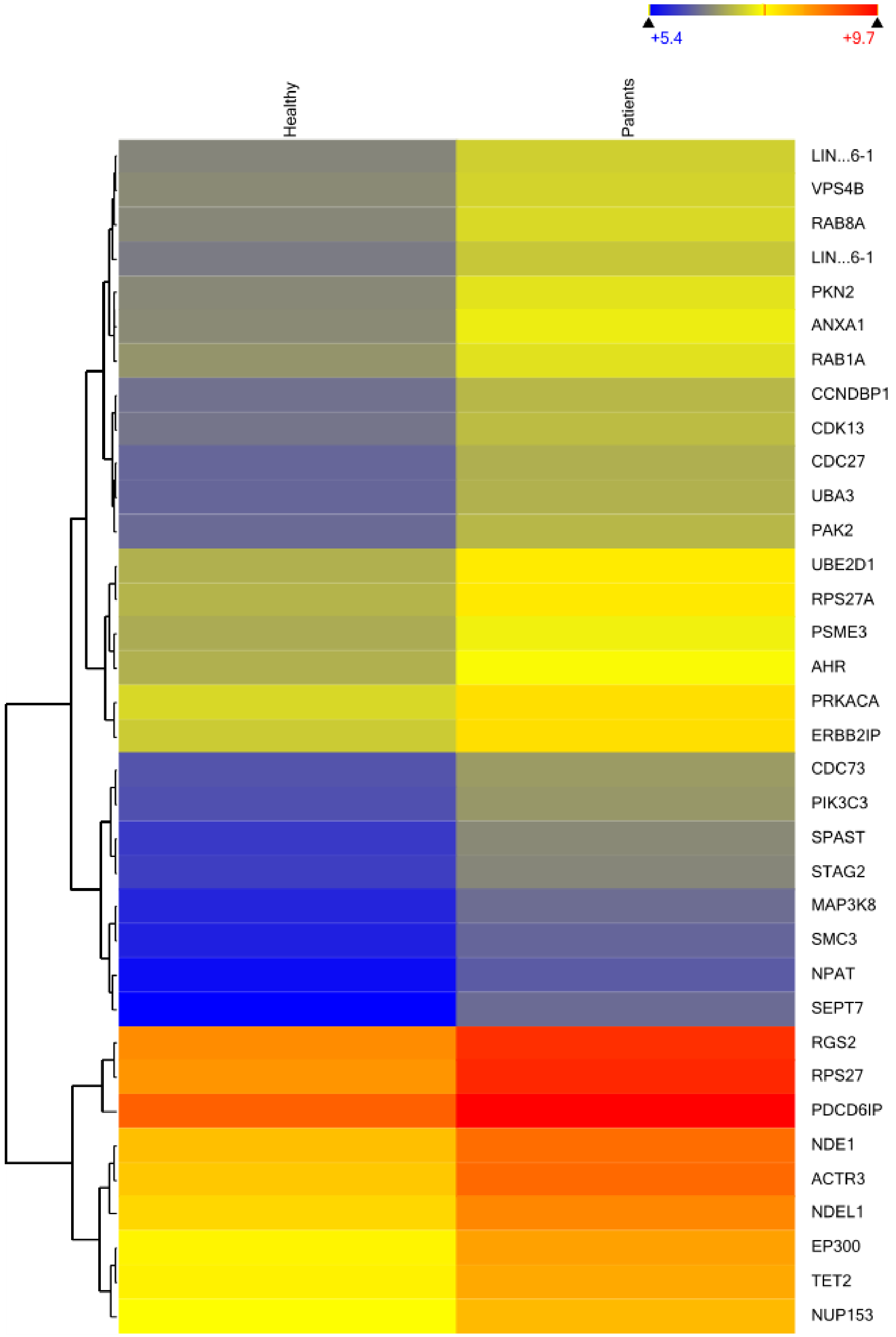

1.4 Figure S4: Immunohistochemical analysis of S100A12 expression. The percentages of positive cells for S100A12 for stage and grade groups in bladder cancer tissue array.

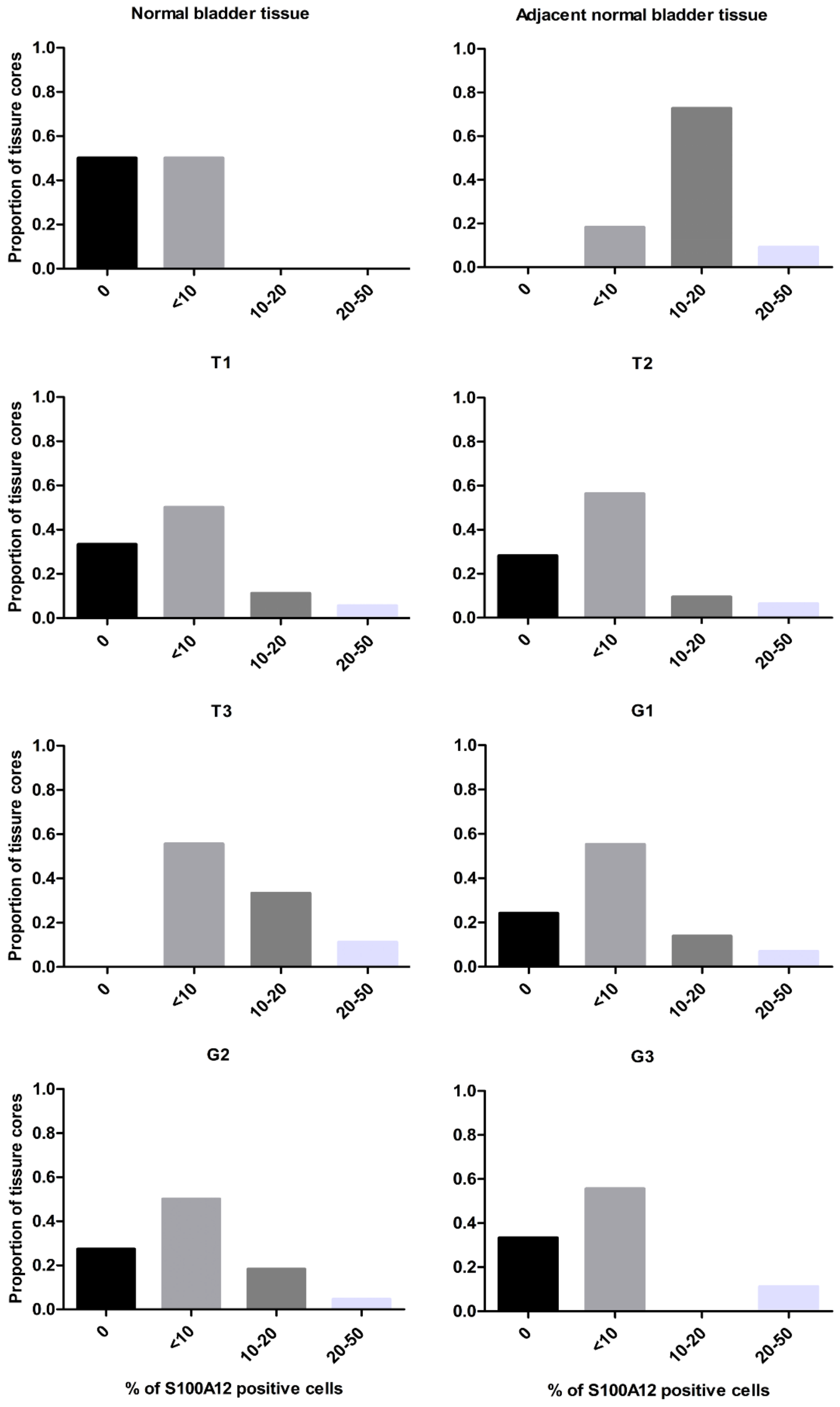

1.5 Figure S5: A ROC analysis of S100A12 and EN2 as combination using CombiROC method.

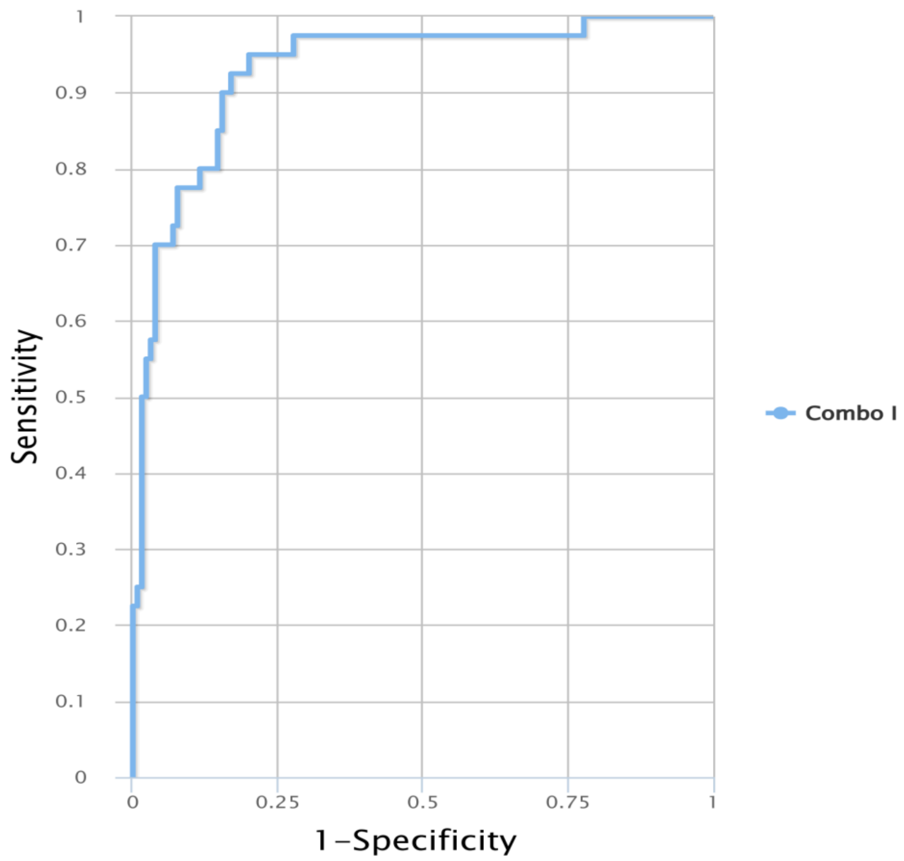

2 Supplementary Tables

2.1 **Table S1:** Primers used in the qPCR analysis.

Gene Symbol	Type	Sequence (5'-3')	Amplicon size [bp]
S100A12	Forward	GCAAACACCATCAAGAATATCAA	119
	Reverse	GCAATGGCTACCAGGGAT A	
S100A8	Forward	GCCAAGCCTAACCGCTATAA	82
	Reverse	CCCACCAGGTCTTCTGAAAG	
NAMPT	Forward	TAT TGC CTT CGG TTC TGG TG	139
	Reverse	TTT GTT GGG ATC AGC AAC TG	
JUP	Forward	AAC CAG GAG AGC AAG CTG AT	128
	Reverse	CAC ACG GAT AGC ACC TTG AG	
KLRF1	Forward	ATG ACC AAC TTG AAA TGG CT	148
	Reverse	GGT CCC TTT ATG AAGAATATCTTTG	
PTGDR	Forward	CTC TGC CCG TAA TTT ATC GC	110
	Reverse	CAC AGA TAG AAA TCG CAA GGC	
GAPDH	Forward	GGAAGGTGAAGGTCGGAGTCA	101
	Reverse	GTCATTGATGGCAACAATATCCACT	

2.2 **Table S2:** Exclusive differentially regulated gene set between recurrent and new positive samples.

Gene Symbol	Gene description	Fold change	p-value
AP001434.2	lincRNA	2.22	<0.001
MYBL1	Myb-related protein A	1.67	0.001
EEF1DP3	pseudogene	1.64	<0.001
RP11-841O20.2	antisense RNA	1.59	<0.001
FGRBP2	fibroblast growth factor binding protein 2	1.59	<0.001
SAMD3	Sterile alpha motif domain-containing protein 3	1.53	<0.001
MME-AS1	antisense RNA	1.52	<0.001
RP11-1143G9.4	antisense RNA	1.51	<0.001

MIR606	miRNA	1.51	<0.001
AFF3	AF4/FMR2 family member 3	-1.50	0.001
GTSF1L	Gametocyte-specific factor 1-like	-1.50	<0.001
ROR1	Inactive tyrosine-protein kinase transmembrane receptor ROR1	-1.51	<0.001
SLC9A7	Sodium/hydrogen exchanger 7	-1.54	0.001
RNU6-517P	snRNA	-1.56	0.001
MIR7641-2	miRNA	-1.59	<0.001
KLHL14	Kelch-like protein 14	-1.63	<0.001
CD200	OX-2 membrane glycoprotein	-1.67	<0.001
FCRL5	Fc receptor-like protein 5	-1.68	<0.001

Data analysis was performed using the software Partek® Genomic Suite (The significant regulated genes were selected using ANOVA test with fold change >1.5 and fdr-corrected p-value <0.05).

2.3 **Table S3:** Differentially regulated gene set in risk group analysis.

**Gene Symbol**	**Gene description**	**Fold change**	**p-value**	**Comparison**
JUP	Junction plakoglobin	1,64	<0,001	High vs. Healthy
RNU6-707P	RNA, U6 small nuclear 707, pseudogene	1,66	0,001	Intermediate vs. Healthy
S100A12	Calcium binding Protein S100A12	1,61	0,001	Intermediate vs. Healthy
CLEC12A	C-type lectin domain family 12 member A	1,59	0,012	Intermediate vs. Healthy
RNU6-237P	RNA, U6 small nuclear 237, pseudogene	1,58	0,002	Intermediate vs. Healthy
IGHV1-2	immunoglobulin heavy variable 1-2	-1,51	0,004	Intermediate vs. Healthy
TRAJ42	T cell receptor alpha joining 42	-1,52	0,022	Intermediate vs. Healthy
TRAJ56	T cell receptor alpha joining 5	-1,53	0,027	Intermediate vs. Healthy
IGKV1-17	immunoglobulin kappa variable 1-17	-1,55	0,012	Intermediate vs. Healthy
TRAJ19	T cell receptor alpha joining 19	-1,56	0,043	Intermediate vs. Healthy

SNORA5A	small nucleolar RNA, H/ACA box 5A	-1,58	0,003	Intermediate vs. Healthy
HIST1H3H	histone H3.1	-1,58	0,028	Intermediate vs. Healthy
IGKV2-24	immunoglobulin kappa variable 2-24	-1,59	0,020	Intermediate vs. Healthy
TRDJ4	T cell receptor delta joining 4	-1,63	0,0348	Intermediate vs. Healthy
IGHG1	Ig gamma-1 chain C region	-1,63	0,054	Intermediate vs. Healthy
IGLV1-40	immunoglobulin lambda variable 1-40	-1,64	0,011	Intermediate vs. Healthy
TRDV1	T cell receptor delta variable 1	-1,66	0,011	Intermediate vs. Healthy
RNU4ATAC	RNA, U4atac small nuclear (U12-dependent splicing	-1,67	0,057	Intermediate vs. Healthy
KLRC3	NKG2-E type II integral membrane protein	-1,71	0,004	Intermediate vs. Healthy
TRAJ17	T cell receptor alpha joining 1	-1,71	0,001	Intermediate vs. Healthy
TRAJ29	T cell receptor alpha joining 29	-1,80	0,001	Intermediate vs. Healthy
